# Biomarkers for Early Severity Prediction in *Clostridioides difficile* Infection: Current Evidence, Clinical Utility, and Future Directions

**DOI:** 10.3390/medicina62071311

**Published:** 2026-07-07

**Authors:** Bianca Balas-Maftei, Carmen-Elena Florea, Lorena Abudanii, Ioana Adelina Stoian, Constantin Aleodor Costin, Maria Grigoriu, Erika Irimie-Baluta, Oana-Manuela Sandu, Alexandra Rotaru, Carmen Manciuc

**Affiliations:** 1 Grigore T. Popa University of Medicine and Pharmacy, 700115 Iasi, Romania; bianca.balas-maftei@umfiasi.ro (B.B.-M.);; 2 Department of Infectious Diseases, “Sfânta Parascheva” Clinical Hospital for Infectious Diseases, 700116 Iași, Romania

**Keywords:** *Clostridioides difficile* infection, CDI, severity, biomarkers, calprotectin, lactoferrin

## Abstract

*Clostridioides difficile* infection (CDI) is a leading healthcare-associated infection worldwide, causing significant morbidity, mortality, healthcare burden, and costs. Clinical manifestations range from mild, self-limiting diarrhea to severe, life-threatening complications such as toxic megacolon and septic shock. Early identification of patients at high risk of severe disease is essential to guide clinical decision-making and optimize therapy. This narrative review summarizes recent epidemiological data, current trends, and known risk factors as clinical context for severity prediction and then examines the utility and limitations of biomarkers that may predict CDI severity, including inflammatory, hematological, fecal, renal, and immune-response biomarkers. While some markers are already used in guideline-based assessment or routine clinical practice (e.g., C-reactive protein, white blood cell count, serum creatinine), they have limited specificity. Other markers emerging from CDI research, including procalcitonin, interleukins, and presepsin, may provide complementary prognostic information. The key challenge is not simply to identify additional biomarkers but to determine which biomarkers are clinically useful, at which stage of CDI progression, and in which patients they add value beyond conventional severity criteria. Validated predictive models integrating combinations of these biomarkers with clinical and microbiological data are needed to support early risk stratification and therapeutic decision-making at the time of diagnosis.

## 1. Introduction

*Clostridioides difficile* infection (CDI) remains one of the leading causes of healthcare-associated infectious diarrhea worldwide and represents a major burden for modern healthcare systems. CDI is associated with substantial morbidity, mortality, prolonged hospitalization, and increased healthcare costs, particularly among elderly and vulnerable patient populations. The main risk factors for CDI are summarized in Table 1; they define the patient populations in whom early severity prediction and risk stratification are particularly relevant [1,2].

Recent global and European epidemiological data further confirm the substantial burden of CDI across hospital, community, and long-term care settings (Table 2). These data support the need for improved early severity assessment, particularly in high-risk populations and healthcare environments where CDI-associated morbidity, recurrence, and mortality remain clinically relevant [1,2].

Despite improvements in infection control measures and therapeutic strategies, the global burden of CDI remains considerable, with both healthcare-associated and community-associated cases continuing to represent relevant clinical and epidemiological challenges [1,10,11,16].

The clinical presentation of CDI is highly heterogeneous, ranging from mild self-limiting diarrhea to severe colitis complicated by toxic megacolon, septic shock, multiorgan dysfunction, and death. This broad clinical spectrum makes early identification of patients at risk for severe disease particularly important, as disease severity at presentation strongly influences therapeutic decisions, escalation of care, and clinical outcomes [3,4,17].

Current severity assessment models recommended by major international guidelines, including those of the Infectious Diseases Society of America (IDSA) and the European Society of Clinical Microbiology and Infectious Diseases (ESCMID), rely predominantly on conventional clinical and laboratory parameters such as leukocytosis, serum creatinine elevation, hypotension, ileus, and toxic megacolon. However, these criteria often reflect relatively advanced disease and may inadequately identify early clinical deterioration, particularly in elderly, immunocompromised, or multimorbid patients, in whom inflammatory responses may be attenuated or atypical [5,6,7,18]. Growing evidence suggests that biomarkers reflecting intestinal injury, systemic inflammation, immune activation, and early organ dysfunction may provide additional prognostic value beyond conventional severity criteria. Biomarkers such as C-reactive protein, procalcitonin, fecal calprotectin, lactoferrin, interleukins, and presepsin have emerged as potential tools for improving early severity prediction and risk stratification in CDI. Their integration into clinical decision-making could, if adequately validated, support earlier identification of high-risk patients and facilitate more individualized therapeutic approaches.

This review is structured around the central question of whether adjunctive host-response biomarkers can improve early CDI severity assessment at the time of diagnosis, beyond currently used guideline-based clinical and laboratory criteria. Rather than presenting biomarkers as isolated prognostic variables, this review interprets them within a pathophysiological and clinical-readiness framework, distinguishing markers of local intestinal injury, systemic inflammatory activation, immune response, and organ dysfunction.

The key unmet need is therefore not simply the discovery of additional biomarkers but the development of clinically interpretable models that determine which biomarkers are useful, at which stage of CDI progression, and in which patient populations they provide incremental value beyond conventional severity criteria.

Accordingly, this narrative review evaluates the current evidence regarding biomarkers for early severity prediction in CDI, discusses their clinical utility and limitations, and highlights future directions for biomarker-based risk stratification models that may improve severity assessment at the time of diagnosis beyond currently used guideline-based clinical and laboratory criteria.

## 2. Materials and Methods

A narrative literature review was performed to identify studies evaluating biomarkers with potential relevance for severity prediction and prognostic assessment in *Clostridioides difficile* infection (CDI). PubMed (National Library of Medicine, Bethesda, MD, USA) was searched in February 2026 for articles published during the previous 20 years (2006–2026), using combinations of the following terms: “*Clostridioides difficile*”, “*Clostridium difficile*”, “CDI”, “biomarkers”, “severity”, “prognosis”, “outcome”, “mortality”, “recurrence”, “C-reactive protein”, “procalcitonin”, “fecal calprotectin”, “lactoferrin”, “interleukins”, and “presepsin”. Additional searches were performed for background information on CDI epidemiology, risk factors, diagnostic algorithms, guideline-based severity criteria, and clinical prediction scores. For broader information describing microbiological characteristics, pathogenesis, clinical manifestations, epidemiology, and risk factors, the publication date was not used as a restrictive filter. Both free-text terms and MeSH terms were used when available, particularly for biomarker-specific searches involving lactoferrin and calprotectin. The retrieved results were screened to identify, select, and review studies focusing specifically on biomarkers that may be used to predict CDI severity, prognosis, or clinically relevant outcomes. An updated search was conducted in early June 2026 to identify newly published or newly indexed studies from 2026.

**Figure 1:**
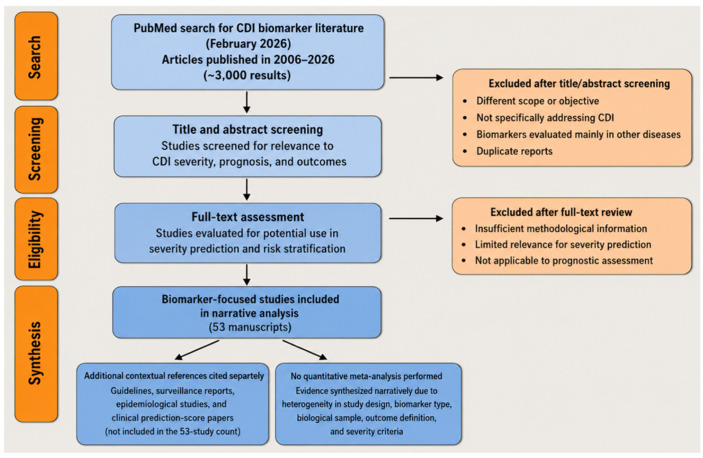
Flow diagram of literature search and study selection.

The initial broad search yielded approximately 3000 results related to *Clostridioides difficile* infection and biomarkers. Following a multi-stage screening process, beginning with title and abstract assessment and followed by full-text evaluation, articles were selected according to their relevance to CDI severity, prognostic assessment, and potential applicability in future risk-stratification models. Articles were excluded if they had a different scope or objective, did not specifically address CDI, evaluated biomarkers primarily in other infectious or non-infectious diseases, lacked sufficient methodological information, or represented duplicate reports. Most excluded articles discussed biomarkers such as lactoferrin or calprotectin in the context of diseases other than CDI or without a focus on CDI severity assessment. Fifty-three manuscripts were considered eligible and included in this biomarker-focused analysis. Guidelines, surveillance reports, epidemiological studies, and clinical prediction-score papers were also cited when necessary to provide clinical context, but they were not considered part of the biomarker-focused eligibility count. Given the heterogeneity in study design, biomarker type, biological sample, outcome definition, and severity criteria, no quantitative meta-analysis was planned or performed; therefore, the evidence was synthesized narratively. The literature search and study selection process is summarized in Figure 1. Figure 1 was prepared using Microsoft PowerPoint for Mac, version 16.110.2 (Microsoft Corporation, Redmond, WA, USA).

## 3. Current Clinical Assessment of CDI Severity

### 3.1. Diagnosis of Clostridioides difficile Infection

The diagnosis of CDI is based on the detection of toxigenic *Clostridioides difficile* or its toxins in stool specimens from symptomatic patients. Current diagnostic approaches include nucleic acid amplification tests (NAATs), enzyme immunoassays (EIAs), and glutamate dehydrogenase (GDH) antigen assays. NAAT offers high sensitivity by detecting toxin-encoding genes, while EIA identifies toxins A and B directly but has lower sensitivity. The GDH assay detects the glutamate dehydrogenase antigen produced abundantly by *C. difficile* and is commonly used as a screening tool [5,16,18].

Current international guidelines discourage indiscriminate standalone testing, particularly NAAT alone in settings without predefined clinical criteria for stool submission. IDSA/SHEA and ESCMID guidance generally support multistep diagnostic algorithms, typically combining GDH and toxin EIA, with NAAT used as a confirmatory method in selected cases. This approach improves diagnostic accuracy by balancing sensitivity and specificity while reducing overdiagnosis related to asymptomatic colonization [5,7,18].

### 3.2. Severity Classification in Current Guidelines

Beyond diagnosis, accurate severity classification remains essential for guiding therapeutic decisions and predicting clinical outcomes. Current IDSA/SHEA and ESCMID guidelines define severe CDI primarily based on conventional clinical and laboratory parameters, including leukocytosis, serum creatinine elevation, and clinical indicators of systemic compromise. In IDSA/SHEA criteria, severe CDI is generally supported by a white blood cell count ≥ 15,000 cells/mm^3^ or serum creatinine > 1.5 mg/dL, while fulminant CDI is characterized by hypotension, shock, ileus, or toxic megacolon and requires urgent escalation of care [5,6,7]. These criteria are widely used in clinical practice because they rely on readily available laboratory and clinical parameters. However, they largely reflect systemic deterioration that may occur later in the disease course, after significant intestinal injury and inflammatory activation have already developed.

### 3.3. Limitations of Current Severity Criteria

Although guideline-based severity criteria provide a practical framework for clinical decision-making, they present important limitations. Leukocytosis and serum creatinine elevation are relatively nonspecific markers and may be influenced by multiple coexisting conditions such as sepsis, chronic kidney disease, corticosteroid therapy, or malignancy. Moreover, elderly and immunocompromised patients may exhibit blunted inflammatory responses, potentially delaying recognition of severe disease [8,9,13,19].

Several clinical prediction tools have been proposed to improve CDI severity assessment, including bedside scores such as ATLAS and other severity or complication prediction models [20]. Although these tools incorporate readily available clinical and laboratory variables and may support risk stratification at diagnosis, their performance has been inconsistent across external validation cohorts [21,22]. Recent comparative validation studies showed that published CDI severity scores have only modest and variable predictive ability, with no single model demonstrating sufficiently robust and reproducible accuracy across different populations and clinical settings [21,22]. These findings support the need for adjunctive biomarkers that could complement, rather than replace, existing clinical criteria and improve early identification of patients at risk for adverse outcomes.

This limitation has generated growing interest in adjunctive biomarkers capable of identifying high-risk patients earlier in the disease course, before overt organ dysfunction develops. Biomarkers reflecting local intestinal injury, systemic inflammatory burden, and immune activation may offer additional prognostic value and improve early risk stratification beyond conventional clinical and laboratory severity criteria.

## 4. Pathophysiological Framework of Severe CDI Progression

The progression of CDI from colonization and mild disease to fulminant colitis is driven by a complex interaction between pathogen virulence, host immune response, and the extent of tissue injury. Disease severity does not result from a single pathological event but rather from a dynamic continuum involving progressive mucosal damage, local and systemic inflammation, and ultimately organ dysfunction. Understanding these sequential stages is essential for interpreting the clinical relevance of different biomarker classes [4,16,23].

### 4.1. Local Intestinal Injury

The initial stage of severe CDI progression is characterized by disruption of the intestinal epithelial barrier following colonization by toxigenic C. difficile strains. Disease pathogenesis is primarily mediated by toxin A and toxin B, which induce epithelial damage through cytoskeletal disruption, increased intestinal permeability, and activation of local inflammatory cascades. Hypervirulent strains such as ribotype 027 (NAP1/BI/027), which may additionally produce binary toxin, have been associated with increased toxin production, more extensive mucosal injury, and higher mortality rates [24,25,26,27].

### 4.2. Systemic Inflammatory Response

As intestinal injury progresses, local inflammation may evolve into a systemic inflammatory response. Activated immune cells release proinflammatory mediators, including interleukins, acute-phase reactants, and other cytokines that contribute to escalating disease severity. This phase is often accompanied by leukocytosis and rising inflammatory biomarkers such as C-reactive protein and procalcitonin. Biomarkers measured during this stage may provide earlier prognostic information than traditional severity criteria by identifying patients with rapidly intensifying inflammatory responses before overt clinical deterioration becomes evident [27,28,29,30].

### 4.3. Organ Dysfunction and Clinical Deterioration

In advanced disease, persistent inflammation and toxin-mediated injury may lead to systemic compromise and organ dysfunction. Renal impairment, hypoalbuminemia, elevated lactate, hypotension, ileus, and toxic megacolon typically reflect this late stage of disease progression and are associated with poor outcomes, including intensive care admission and mortality. Importantly, many conventional severity markers currently used in clinical guidelines predominantly capture this advanced stage, which may limit opportunities for earlier therapeutic intervention [8,9,26,31].

**Figure 2:**
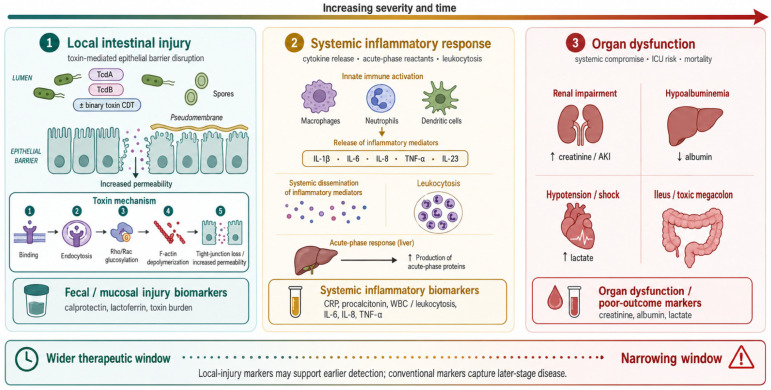
Pathophysiological framework of severe CDI progression and biomarker dynamics. CDI severity may increase from toxin-mediated epithelial injury and mucosal inflammation to systemic inflammatory activation and, in advanced cases, organ dysfunction. Fecal biomarkers may predominantly reflect local intestinal injury, circulating inflammatory biomarkers may reflect systemic immune activation, and conventional laboratory and clinical markers may capture later-stage physiological deterioration. This framework supports the use of biomarkers as adjunctive tools for earlier risk stratification rather than as replacements for guideline-based severity criteria. CDI: *Clostridioides difficile* infection; TcdA: toxin A; TcdB: toxin B; CDT: *Clostridioides difficile* transferase/binary toxin; IL: interleukin; TNF-α: tumor necrosis factor alpha; CRP: C-reactive protein; WBC: white blood cell count; AKI: acute kidney injury; ICU: intensive care unit.

This pathophysiological framework suggests that different biomarkers may offer prognostic value at distinct stages of disease progression. Biomarkers reflecting local intestinal injury may improve early recognition of patients with biologically active and potentially severe disease, whereas systemic inflammatory and organ dysfunction markers may better predict imminent clinical deterioration and short-term adverse outcomes (Figure 2).

Importantly, these stages should not be interpreted as strictly separate events, but rather as overlapping biological processes that may coexist at the time of diagnosis. Therefore, a single biomarker is unlikely to capture the full spectrum of CDI severity. A combined approach integrating fecal markers of mucosal injury, circulating inflammatory biomarkers, and conventional clinical or laboratory severity criteria may provide a more comprehensive framework for early risk stratification [32].

## 5. Biomarker Classes for Early Severity Prediction

A wide range of biomarkers has been investigated for their ability to predict severe CDI and adverse clinical outcomes. From a pathophysiological perspective, these biomarkers may reflect distinct biological processes, including systemic inflammation, intestinal mucosal injury, immune activation, and early organ dysfunction. For clinical interpretation, biomarkers can be broadly classified according to their biological significance and potential applicability in routine practice [3,4]. An overview of the major biomarker classes, their biological interpretation, clinical utility, and main limitations is provided in Table 3. This classification also allows biomarkers to be interpreted according to their clinical readiness, ranging from routinely available laboratory parameters to investigational molecular and microbiome-based approaches.

### 5.1. Routine Laboratory Biomarkers

Routine laboratory parameters remain the most widely available biomarkers used in the initial assessment of CDI severity. Their major advantage lies in universal availability, low cost, and rapid turnaround time, making them particularly useful in emergency and inpatient settings. However, despite their practicality, most are limited by suboptimal specificity and may be influenced by multiple comorbid conditions. Leukocytosis remains one of the most established markers of severe CDI and is incorporated into both IDSA and ESCMID severity criteria. Elevated white blood cell counts reflect systemic inflammatory activation and correlate with increased disease severity, complications, and mortality risk. However, leukocytosis lacks specificity and may be influenced by concurrent infections, corticosteroid therapy, hematologic disorders, or immunosuppression [5,7,8,19]. C-reactive protein (CRP) is a widely used acute-phase reactant that increases in response to systemic inflammation. Several studies have associated elevated CRP levels with severe CDI, prolonged hospitalization, and adverse outcomes. CRP may offer additional prognostic value when interpreted alongside conventional severity markers, although elevated levels are not specific to CDI-related inflammation [33,37].

Serum creatinine serves as an indirect marker of renal dysfunction and systemic compromise. Elevated creatinine is currently integrated into guideline-based severity classification and often reflects dehydration, hypoperfusion, or evolving multiorgan dysfunction. Although clinically valuable, creatinine elevation generally represents later disease progression rather than early severity escalation [5,7,37]. Hypoalbuminemia has been associated with severe disease, increased recurrence risk, and poorer outcomes in CDI. Low serum albumin may reflect systemic inflammation, malnutrition, capillary leak, or chronic illness burden. Because albumin integrates both acute and chronic patient vulnerability, it may complement inflammatory biomarkers during risk assessment [2,8,19]. Serum lactate is particularly relevant in advanced disease, where tissue hypoperfusion and systemic compromise become prominent. Elevated lactate has been associated with fulminant CDI, shock, and mortality; similar to creatinine, it primarily reflects late-stage clinical deterioration rather than early disease progression [31,49].

### 5.2. Systemic Inflammatory Biomarkers

Systemic inflammatory biomarkers may offer earlier prognostic information than conventional severity criteria by reflecting the intensity of the host inflammatory response before overt clinical deterioration develops. These markers have attracted increasing attention as potential adjunctive tools for identifying patients at risk of rapid progression toward severe or fulminant CDI [28,30].

Procalcitonin (PCT) is a biomarker commonly associated with bacterial infection and systemic inflammatory activation. In CDI, elevated PCT levels have been linked to increased disease severity, systemic complications, and worse clinical outcomes. Compared with conventional inflammatory markers, PCT may provide additional prognostic information in distinguishing patients with significant inflammatory burden, although current evidence remains limited by small study populations and heterogeneity in cutoff values [28,34,35,36].

Interleukins represent key mediators of the inflammatory cascade and may reflect early immune activation during severe CDI. Elevated levels of proinflammatory cytokines such as interleukin-6 (IL-6) and interleukin-8 (IL-8) have been associated with more severe disease, increased toxin-mediated inflammation, and poorer clinical outcomes. These cytokines may capture upstream inflammatory activation that is not fully reflected by routine laboratory markers, potentially improving early severity prediction [29,30]. IL-8 plays a central role in neutrophil recruitment and amplification of mucosal inflammation, thereby contributing to intestinal tissue injury during severe CDI. In contrast, IL-6 acts as a key mediator of systemic acute-phase signaling and stimulates hepatic synthesis of proteins such as CRP. Because these cytokines reflect upstream inflammatory activation, they may provide biologically relevant prognostic information; however, their added value over routine laboratory parameters remains insufficiently validated [50].

Additional cytokines, including interleukin-1 and interleukin-4, have also been investigated as potential biomarkers of disease progression. However, the available evidence remains limited and inconsistent, partly due to interstudy variability in assay methodology, patient populations, and severity definitions. Despite their biological plausibility, systemic inflammatory biomarkers remain limited by restricted availability, assay cost, and lack of standardized thresholds for routine clinical use. Further prospective studies are needed to determine whether these markers can reliably improve severity stratification beyond conventional laboratory parameters.

### 5.3. Intestinal Inflammatory Biomarkers

Unlike systemic biomarkers, fecal inflammatory markers may provide more direct insight into local intestinal injury and mucosal inflammation. Because CDI is fundamentally a toxin-mediated colonic disease, biomarkers reflecting local inflammation may theoretically detect severe disease earlier than markers of systemic compromise [38,51]. Fecal calprotectin is a calcium-binding protein released predominantly by activated neutrophils and serves as a marker of intestinal inflammation. Elevated fecal calprotectin levels have been associated with severe CDI, greater toxin burden, and increased inflammatory activity within the colon. Its major advantage lies in its ability to reflect local disease activity, although interpretation may be complicated by other gastrointestinal inflammatory conditions [38,39,40,41,51].

Lactoferrin is an iron-binding glycoprotein released by activated neutrophils during inflammatory processes. Increased fecal lactoferrin concentrations have been correlated with severe colonic inflammation and higher CDI severity in several studies. Similar to calprotectin, lactoferrin offers valuable information regarding mucosal inflammatory burden but lacks specificity for CDI [41,42,43,51]. Although fecal biomarkers offer important pathophysiological insights, their routine clinical utility remains limited by assay availability, processing requirements, and insufficient validation in large prospective cohorts.

### 5.4. Emerging Biomarkers

Emerging biomarkers aim to improve severity prediction by capturing biological processes not adequately reflected by routine laboratory testing. These include novel sepsis-associated markers, microbiome-derived signatures, and advanced molecular approaches.

Presepsin, a soluble CD14 subtype generated during monocyte activation, has emerged as a promising biomarker for systemic infection and sepsis. In CDI, elevated presepsin levels may reflect excessive immune activation and have been associated with severe disease and poorer outcomes in preliminary studies. Its biological relevance lies in its ability to reflect monocyte and macrophage activation during systemic inflammatory responses [44,45,46,47].

Compared with traditional inflammatory markers, presepsin has shown potential advantages in certain sepsis cohorts due to its rapid rise during early immune activation. Because CDI severity may evolve through systemic inflammatory pathways resembling sepsis, presepsin may theoretically identify high-risk patients before classical organ dysfunction markers become abnormal. However, interpretation may be complicated in patients with impaired renal function, as circulating presepsin concentrations can be influenced by reduced clearance [48].

Advanced approaches such as proteomics, metabolomics, and microbiome profiling may further improve future risk prediction by identifying complex molecular signatures associated with severe CDI. Reduced microbial diversity, altered bile acid metabolism, and strain-specific toxin expression patterns have all been implicated in disease severity and recurrence. Such approaches may eventually allow personalized risk stratification by integrating microbial ecosystem disruption with host inflammatory responses [14,15].

While emerging biomarkers offer substantial research potential, their implementation in routine clinical practice remains limited by cost, technical complexity, and insufficient external validation. At present, these approaches should be considered investigational rather than clinically established.

From a clinical-readiness perspective, these biomarker classes should not be interpreted as equivalent. Routine laboratory biomarkers currently have the highest applicability because they are inexpensive, rapidly available, and already integrated into clinical workflows, although their specificity for CDI severity is limited [5,7,8,19]. Systemic inflammatory biomarkers, particularly PCT and selected cytokines, may provide additional prognostic information but remain constrained by heterogeneous cutoff values and limited external validation [28,30,34,35,36,50]. Fecal inflammatory biomarkers are biologically attractive because they reflect mucosal injury more directly, but their routine use is limited by assay availability, turnaround time, and lack of standardized thresholds [41,42,43,51]. Emerging molecular, microbiome-based, and sepsis-associated biomarkers currently remain investigational and should be regarded as hypothesis-generating tools for future integrated risk-stratification models rather than standalone predictors of severe CDI [14,15,44,48].

## 6. Host-Response Biomarkers Versus Pathogen Virulence Factors

Accurate severity prediction in CDI requires the integrated assessment of both host-related and pathogen-related determinants. Disease progression results not only from the host inflammatory response but also from pathogen virulence characteristics that influence toxin production, mucosal injury, and overall clinical aggressiveness. Distinguishing these two dimensions provides a more comprehensive framework for understanding disease severity and for interpreting why biomarkers measured in the host may not always correlate directly with microbiological markers of strain virulence [4,16,23]. Host-response biomarkers primarily reflect the magnitude of systemic inflammation, immune activation, and organ dysfunction triggered by the infection. Biomarkers such as C-reactive protein, procalcitonin, interleukins, lactate, and serum creatinine provide indirect information regarding the host’s physiological response to toxin-mediated injury. Their prognostic value lies in identifying patients with exaggerated inflammatory responses or early systemic compromise [28,30,33].

In contrast, pathogen virulence markers reflect strain-related features that may increase the capacity of C. difficile to induce severe disease. Hypervirulent strains such as ribotype 027 (NAP1/BI/027) are associated with increased toxin production, fluoroquinolone resistance, greater mucosal damage, higher recurrence rates, and increased mortality in several epidemiological settings. The presence of binary toxin and a higher toxin burden may further contribute to disease severity by intensifying intestinal injury and inflammatory activation [12,24,25,26,51]. However, pathogen-related markers are not always available at the time of diagnosis and may not fully explain clinical heterogeneity, because outcomes are also strongly influenced by age, comorbidities, immune status, renal function, and the intensity of the host inflammatory response.

Neither host-response biomarkers nor pathogen virulence markers alone appear sufficient to fully predict disease trajectory. A combined framework integrating both host susceptibility and pathogen virulence may offer superior prognostic performance and represents a promising direction for future severity prediction models in CDI. In this context, host-response biomarkers may be more immediately applicable for early bedside risk stratification, whereas pathogen virulence markers may provide complementary information for epidemiological surveillance, outbreak investigation, and the refinement of future prognostic models.

## 7. Clinical Utility and Biomarker Readiness

Although numerous biomarkers have shown potential prognostic value in CDI, their applicability in routine clinical practice varies substantially (Table 4). Clinical utility depends not only on predictive performance but also on availability, turnaround time, assay standardization, and cost-effectiveness [3,4].

From a practical clinical perspective, biomarker utility depends on the stage of evaluation and the clinical question being addressed. At initial presentation, readily available biomarkers such as leukocyte count, serum creatinine, CRP, and albumin are among the most useful parameters for rapid bedside risk assessment and for identifying patients who already meet guideline-based severity criteria. However, these parameters may inadequately capture patients with evolving severe disease who have not yet developed overt organ dysfunction [5,7].

In situations where conventional markers provide discordant or borderline results, adjunctive biomarkers may improve decision-making. For example, elevated procalcitonin may suggest a more intense systemic inflammatory response, while elevated or rising lactate should raise concern for evolving systemic compromise rather than early uncomplicated disease. In such contexts, these biomarkers may justify closer monitoring, escalation of therapy, or earlier intensive care evaluation. Similarly, fecal inflammatory biomarkers may offer additional insight in selected cases where the degree of local intestinal inflammation appears disproportionate to systemic laboratory abnormalities.

Routine biomarkers such as white blood cell count, C-reactive protein, serum creatinine, and albumin currently offer the highest clinical readiness due to universal availability, low cost, and rapid laboratory processing. These markers remain highly practical for initial severity assessment despite their limited specificity. Their widespread accessibility makes them the most feasible biomarkers for routine clinical implementation at present [5,33,37].

Biomarkers such as procalcitonin and lactate show promising adjunctive value by potentially improving early risk stratification beyond conventional criteria. Procalcitonin may be more informative for systemic inflammatory activation, whereas lactate is particularly relevant for identifying evolving or advanced physiological compromise. However, broader validation is required before they can be routinely incorporated into severity prediction algorithms [28,31,34,36,49].

Less established biomarkers, including interleukins, presepsin, fecal calprotectin, and lactoferrin, remain largely research-oriented or selectively applicable. Despite encouraging preliminary evidence, limited availability, higher costs, and lack of standardized cutoff values currently restrict their routine clinical implementation [30,38,44,45,51].

Rather than replacing conventional severity criteria, biomarkers are more likely to function as complementary tools within multimodal clinical assessment [23,42]. Their greatest value may lie in identifying patients at risk of deterioration earlier than traditional criteria allow, thereby enabling more timely therapeutic interventions [5,8,9].

At present, no single biomarker demonstrates sufficient accuracy to independently predict CDI severity across diverse clinical settings. The most promising future approach may involve multimarker models integrating complementary biomarkers with clinical and microbiological parameters.

## 8. Challenges and Future Directions

Despite growing interest in biomarker-based severity prediction, several important barriers continue to limit the integration of these markers into routine clinical practice. Current evidence remains heterogeneous in terms of study design, patient selection, severity definitions, and biomarker thresholds, making direct comparison across studies difficult [3,4].

One of the major challenges is the lack of standardized cutoff values for most biomarkers. Different studies have used variable thresholds for markers such as C-reactive protein, procalcitonin, and fecal calprotectin, resulting in inconsistent estimates of prognostic performance. This heterogeneity complicates clinical interpretation and limits external reproducibility [34,37,38,51].

Practical limitations also influence clinical applicability. While routine biomarkers are widely available, more advanced inflammatory or molecular biomarkers may require specialized laboratory infrastructure, increased costs, and longer turnaround times, limiting accessibility in many healthcare settings [30,44,51].

Another major limitation is the lack of prospective multicenter validation. Many available studies are retrospective, involve relatively small cohorts, or originate from single institutions, reducing generalizability across diverse patient populations and healthcare systems [8,9,17,19]. In addition, few studies have directly compared novel biomarkers with currently used guideline-based severity criteria or established clinical prediction scores, making it difficult to determine their true incremental prognostic value [23,24,25].

Future research should prioritize the development of multimarker prediction models that integrate biomarkers reflecting complementary biological pathways, including intestinal injury, systemic inflammation, and organ dysfunction. Combining biomarker panels with clinical variables, microbiological characteristics, and pathogen virulence factors may improve predictive accuracy compared with isolated markers [23,24,25]. However, such models should be designed to remain clinically interpretable, feasible in routine practice, and externally validated before implementation.

Emerging computational approaches, including machine learning and artificial intelligence-based prediction models, may further enhance severity stratification by identifying complex nonlinear relationships between biomarkers and clinical outcomes. Nevertheless, these approaches should be considered exploratory at present, because their clinical value depends on transparent model development, adequate sample size, external validation, and demonstration of added benefit over simpler bedside tools. Such approaches may enable more individualized risk assessment and support precision medicine strategies in CDI management.

Ultimately, large prospective multicenter studies are required to validate biomarker-driven prediction models and determine whether their implementation can meaningfully improve therapeutic decision-making and patient outcomes in routine clinical practice. Until such evidence becomes available, biomarkers should be viewed as adjunctive tools that may refine, but not replace, conventional clinical assessment of CDI severity.

## 9. Conclusions

Early severity prediction remains a critical challenge in the management of *Clostridioides difficile* infection. Although current guideline-based severity assessment provides a practical clinical framework, conventional criteria often identify severe disease only after substantial systemic deterioration has occurred.

A wide range of biomarkers reflecting intestinal injury, systemic inflammation, immune activation, and organ dysfunction have shown potential for improving early risk stratification. However, no single biomarker currently demonstrates sufficient accuracy or validation to independently guide clinical decision-making across diverse patient populations.

The most promising future direction lies in multimarker prediction models that integrate biomarker panels with clinical and microbiological parameters. Such approaches may enable earlier identification of high-risk patients and support more individualized therapeutic strategies. However, their clinical implementation will require prospective multicenter validation and demonstration of added value beyond currently used guideline-based severity criteria. The future of CDI severity prediction is unlikely to depend on a single universal biomarker but rather on staged, multimodal models that integrate host-response biomarkers, conventional laboratory criteria, clinical vulnerability, and pathogen-related information. Such an approach may shift severity assessment from late recognition of organ dysfunction toward earlier identification of biologically high-risk disease.

## Figures and Tables

**Table 1:** Main risk factors for *Clostridioides difficile* infection and their clinical impact.

Risk Factor	Mechanism/Context	Clinical Impact	References
Antibiotic exposure	Broad-spectrum antibiotics including (fluoroquinolones, clindamycin, third-Generation cephalosporins, and carbapenems)	Strongest modifiable risk factor; up to 7–10-fold increased risk, depending on antibiotic class and exposure window	[3,4,5,6,7,8]
Advanced age (>65 years)	Age-related decline in gut microbiome diversity, immune senescence, increased comorbidities, and frequent healthcare contact	Accounts for >95% of CDI-related deaths globally	[4,9,10,11]
Prior hospitalization(s)	Prolonged hospital stays, ICU admissions, surgical procedures, and invasive devices	HA-CDI incidence 3–5× higher than CA-CDI	[1,4,10,11,12]
Long-term care facility residence	Shared environment, frequent use of antibiotics, and immunocompromised residents	Highest incidence: 44.24 cases/10,000 patient-days	[1,4]
Proton pump inhibitor (PPI) use	Reduced gastric acidity facilitating *C. difficile* spore survival and colonization	~25% of adults use PPIs globally; associated with CDI and rCDI	[3,4]
Immunosuppression and/or malignancy	Chemotherapy, corticosteroids, hematological malignancies, and other conditions and treatments that impair immune defenses	Independent predictor of CDI mortality in multivariate analysis	[8,9,13]
Prior CDI episode(s)	Disrupted microbiome, residual spores, and impaired mucosal immunity	25–35% recurrence risk; each recurrence raises mortality 2.7% to 25%	[2,3,4,7]
COVID-19 infection	Dysbiosis, frequent antibiotic use in COVID management, and ICU admission	HA-CDI incidence 3× higher in COVID-19 patients	[3,4]

HA-CDI: healthcare-associated *Clostridioides difficile* infection; CA-CDI: community-associated *Clostridioides difficile* infection; rCDI: recurrent *Clostridioides difficile* infection; ICU: intensive care unit; PPI: proton pump inhibitor; COVID-19: coronavirus disease 2019.

**Table 2:** Summary of key global and European epidemiological data for *C. difficile* infection.

Setting/Indicator	Value	Region	References
Hospital-onset/healthcare-facility-associated CDI	5.31 cases/1000 admissions 5.00 cases/10,000 patient-days	Global (59 studies)	[1]
Long-term care facilities	44.24 cases/10,000 patient-days	Global	[1]
Europe (HA-CDI)	2.58 cases/10,000 patient-days	EU/EEA	[14]
Europe (CA/UA-CDI)	1.35 cases/1000 admissions	EU/EEA	[14]
Recurrence rate (community-associated)	16.22%	Global	[1]
All-cause mortality	16.05%	Global meta-analysis	[1]
Age-standardized mortality (2021)	0.19/100,000 population	Global (GBD 2021)	[15]
30-day mortality (CDI)	8.32%	Global meta-analysis	[1]
1-year mortality in patients ≥65 years	35–45%	USA Medicare	[12]

CDI: *Clostridioides difficile* infection; HA-CDI: healthcare-associated CDI; CA/UA-CDI: community-associated or unknown-association CDI; EU/EEA: European Union/European Economic Area; GBD: Global Burden of Disease; USA: United States of America.

**Table 3:** Biomarkers for predicting severity in *Clostridioides difficile* infection.

Biomarker Types	Examples	Indicative of	Clinical Utility	Limitations	References
Systemic inflammatory markers	CRP; procalcitonin	Systemic inflammation and inflammatory burden	Predict severity, ICU risk	Low specificity, also elevated in many other inflammatory or infectious conditions	[28,30,33,34,35,36]
Hematological parameters	Leukocytosis, WBC count, neutrophil count	Inflammatory response and bone marrow activation	Key component of current guideline-based severity stratification	May be normal in elderly or immunocompromised patients with severe disease	[5,6,7,8,19,37]
Renal/Nutritional markers	Creatinine, albumin	Organ dysfunction and patient vulnerability	Creatinine: severity criterion Albumin: predictor of mortality and recurrence	Levels also influenced by comorbidities (e.g., chronic kidney disease, liver disease, malnutrition)	[5,6,7,8,9,37]
Fecal biomarkers	Calprotectin, lactoferrin	Local intestinal inflammation and neutrophil activation	Reflect mucosal inflammatory activity and may complement systemic markers	Elevated also in other intestinal inflammatory conditions (e.g., IBD)	[12,38,39,40,41,42,43]
Emerging or investigational biomarkers in CDI	Presepsin, IL-6, IL-8, molecular and microbiome-based signatures	Immune activation and systemic inflammatory response, and host–microbiome disruption	Potential early predictors of severe disease and systemic complications	Limited availability and insufficient validation in large prospective cohorts	[14,15,29,30,44,45,46,47,48]

CDI: *Clostridioides difficile* infection; CRP: C-reactive protein; WBC: white blood cell count; ICU: intensive care unit; IBD: inflammatory bowel disease; IL: interleukin.

**Table 4:** Proposed clinical-readiness ranking of biomarkers for CDI severity prediction.

Readiness Level	Biomarker Category	Examples	Current Interpretation	References
High clinical readiness	Routine laboratory biomarkers	WBC count, serum creatinine, CRP, albumin	Widely available, inexpensive, rapidly processed, and already integrated into routine clinical workflows; useful for initial severity assessment, although limited by low specificity	[5,7,8,19,33,37]
Moderate clinical readiness	Adjunctive systemic biomarkers	Procalcitonin, lactate	May support risk stratification in selected patients, particularly when conventional criteria are borderline or discordant; broader CDI-specific validation is still needed	[28,31,34,36,49]
Limited clinical readiness	Fecal and cytokine biomarkers	Fecal calprotectin, lactoferrin, IL-6, IL-8	Biologically relevant because they reflect mucosal inflammation or upstream immune activation, but routine implementation is limited by assay availability, turnaround time, cost, and lack of standardized thresholds	[12,30,38,41,42,43,50]
Investigational	Emerging molecular and microbiome-based approaches	Presepsin, proteomic profiles, metabolomic signatures, microbiome-derived markers	Promising for future integrated prediction models, but currently insufficiently validated for routine clinical decision-making	[14,15,44,45,46,47,48]

WBC: white blood cell count; CRP: C-reactive protein; IL: interleukin.

## Data Availability

No new data were created or analyzed in this study. Data sharing is not applicable to this article.

## Abbreviations

AKI	Acute kidney injury
ATLAS	Age, Treatment with systemic antibiotics, Leukocyte count, Albumin, Serum creatinine
CA-CDI	Community-associated *Clostridioides difficile* infection
CDI	*Clostridioides difficile* infection
CRP	C-reactive protein
EIA	Enzyme immunoassay
ESCMID	European Society of Clinical Microbiology and Infectious Diseases
GDH	Glutamate dehydrogenase
HA-CDI	Healthcare-associated *Clostridioides difficile* infection
IBD	Inflammatory bowel disease
ICU	Intensive care unit
IDSA	Infectious Diseases Society of America
IL	Interleukin
NAAT	Nucleic acid amplification test
PCT	Procalcitonin
PPI	Proton pump inhibitor
rCDI	Recurrent *Clostridioides difficile* infection
SHEA	Society for Healthcare Epidemiology of America
WBC	White blood cell
